# Potentiation a Consequence of the Law of Similia, and *Vice Versa*, from the Hahnemannian Standpoint

**Published:** 1884-11

**Authors:** B. Fincke

**Affiliations:** Brooklyn, N. Y.


### HOMŒOPATHIC PHYSICIAN.

A MONTHLY JOURNAL OF MEDICAL SCIENCE.

"If our school ever gives up the strict inductive method of Hahnemann, we are lost, and deserve only to be mentioned as a caricature in the history of medicine."—CONSTANTINE HERING.

Vol. IV. NOVEMBER, 1884. No. 11.

### POTENTIATION A CONSEQUENCE OF THE LAW OF SIMILIA, AND VICE VERSA, FROM THE HAHNEMANNIAN STANDPOINT.

B. FINCKE, M. D., BROOKLYN, N. Y.

Hahnemann says (*Organon*, 5th ed., sec. 10): "The material organism without a vital force is not capable of any sensation, of any activity, of any self-preservation, only the immaterial essence which vivifies the material organism in the healthy and sick state (the vital force) imparts to it all sensation and actuates its vital functions."

This is the fundamental proposition which must be accepted by every homœopathician if he means to find any sense in Homœopathy at all. If he cannot accept it on account of the general rejection of a vital force at the hands of the modern physicists, he must try to free himself from the tenets of a school which, essentially, has nothing to do with the science and art of healing. Their limited conception of force may be sufficient for physics and chemistry and their practical purposes, but it falls short of the requirements of the science which we cultivate in order to cure our patients *cito, tuto et jucunde.*

From this acceptance of the vital force follows necessarily the conception of disease expressed in sec. 12: "Only the morbidly distuned vital force brings forth the diseases."

Hence diseases are not recognized but as (sec. 6) "changes of the state of mind and body noticeable externally by the senses as symptoms, deviations from the previously healthy state of the patient which he himself feels, which the bystanders and the physicians observe upon him. All these observable signs represent the disease in its whole compass."

For this reason (sec. 17) "the healing artist has only to take away the whole complex of the symptoms in order to remove and annihilate at the same time the inner change, *i. e.*, the morbid distunement of the vital force, therefore the totality of the disease—the disease itself—after which health is restored."

This is done (sec. 25) "by remedies which in their action upon the healthy human organism have been proved to be able to produce the greatest number of similar symptoms in properly potentiated and lessened doses."

This rests (sec. 26) upon the homœopathic general law, "that a weaker dynamic affection in the living organism is extinguished permanently by a stronger one, if this (different in kind) is very similar to it in its utterance."

The probable scientific reason for this law, which is demonstrated and verified in every case of healing, is (sec. 28) in that "when the homœopathic remedy is given, a somewhat stronger artificial disease is created in the diseased organism by the positive action rendering the opposite natural disease negative and extinguishing it. This substitution of the artificial to the natural disease, by which it is superseded, rouses the energy of the vital force to overcome the superior strength of the remedy," which, being a mere potency without substance behind it, and only stronger than the disease by a Least Plus (Maupertuis' least action, *Fincke's Least Plus, High Potencies and Homœopathics*, pp. 18, 129), vanishes in these efforts, and the vital force returns to its equilibrium of health without further than the normal functional oscillations. This explanation is so forcible that Trousseau and Schoenlein, two of the greatest physicians who ever lived, accepted it under the name, "Substitutive method of Medicine." But Homœopathy is more.

It is impossible to escape the consequences flowing from these ideas of Hahnemann, which now for seventy-five years have guided his true followers, who in a large practice of so many years found them correct up to the present day.

The fact that cures are also effected by crude medicines and large doses and by various agents and methods standing in the signification of remedies is undeniable, but has nothing to do with the Hahnemannian doctrine of Homœopathy even if the remedy should be homœopathic to the case. They have a different *modus operandi* and do not act like the properly potentiated homœopathic remedy. This question, however, requires a treatment of its own, which is not the object of this paper.

If the remedy in too large a dose overpowers the system in virtue of its homœopathicity to such a degree that its action is not disappearing in the efforts of the vital force to restore the equilibrium, there must result a serious aggravation which jeopardizes the cure by removing the oscillations to farther limits, and the danger is in the weakening of the vital force, which cannot quick enough regain the lost normal equilibrium. Still worse it is if the large dose is not homœopathic to the case. Then the disease present is increased by the totality of the symptoms produced by the allopathic remedy and no recovery is likely to ensue.

This is the reason why Hahnemann, with perfect insight into the nature of the healing process, has, in his later life, after a long and varied experience, insisted upon the doctrine that "in every case whatever a dose should be given which, in the proper way, has been potentiated according to a method growing out of the necessities of homœopathic practice, so that no chemical and physical test can find the least particle of atom of matter in it." *Kleine Schriften*, Vol. II, p. 199.) Why there should be in this appropriate homœopathic medicine still the power or potency to cure, is a question which requires an investigation of its own, and cannot here be entered upon.

The point to be shown here is, that the remedy to be used by the homœopathician for the purpose of cure should always be potentiated at least to such a degree that no matter can be found in it.

This has been shown to be at about the tenth centesimal potency. I have made the spectroscopic test without telescope, and found the limit at the ninth centesimal. Probably with proper microscopical arrangements the limit might be a little further off, but will fall short of the thirtieth centesimal, which Hahnemann in his time recommended as the standard potency. The neurological test by Jaeger's and Fincke's method carries us much higher, nay, with the electro-neurometer any potency may be shown to act upon a suitable person within a few minutes. This, however, is not a physical, but a physiological test, and shows the correctness of Hahnemann's explanation of a fact which has been rejected by the physicists, who cannot see force in anything that is not matter or not connected with matter. But if they would reflect only for a moment and be just, they would have to admit that the potency is actually carried by matter, viz.: the inert vehicle, and that the vital force is actually supported by the nerve-system as the vehicle of its action upon the whole organism. But for that matter it does not follow that the potency contained in a vial and the vital force contained in the body should be matter. This is the vital, spirit-like principle which animates the otherwise inert matter, gives it its quality and property, which it again renders in potentiation.

It is therefore by no means in the option of the homœopathician to select a large or a small dose according to his whim, as he may feel inclined or be biased, but he must, owing to the nature of the homœopathic principle, always use a remedy with no body to it except the inert vehicle, that is, a potency, if he means to make a clean and perfect cure without drawbacks of any kind. This question of the dose, at which so many have unnecessarily excited themselves waiting for its solution, as the Jews for the Messiah, has been closed by Hahnemann long ago, when he wrote his *Organon*, fortieth edition, in 1829, and published his *Chronic Diseases*, 1828–1829, being at that time seventy-four years old.

It is another question: Which is the appropriate potency in each given case? This, of course, will ever be open, and will have to be solved by the physician in attendance.

Now perhaps it might be said, this is all very well, but it is only a deduction from the vital principle, which is not acknowledged by the scholars of the present day, who will accept nothing but what is established beyond any doubt by induction. Therefore, if Homœopathy stands upon the vital principle alone, it of right has no status in the present scientific world, and that is the reason why our adversaries will do no more than pick out the gold nuggets which the patient and long-enduring Homœopathy has brought up from the darkness of human understanding, appropriating them without giving credit where credit is due.

This apparently formidable rejection need not be appalling to the homœopathician. For he knows that before Hahnemann laid down his thoughts on the healing process he had gone through that stage of induction claimed above, which led him to pronounce distinctly the principle from which Homœopathy flows like a river from its fountain head. The elements of this induction, as there is no better one for the exact natural sciences, are found also in the *Organon* from which the above elements of deduction have been quoted.

It has not been sufficiently acknowledged by the scientific body at large that Hahnemann created homœopathics as a complete science of medicine resting upon the sure foundation of induction and deduction. His induction has been accepted as empirical science of therapeutics (Dunham), and as such has done immense service by its practical application and by its influence on the allopathic school. His deduction has been laid by as peculiar to the old man, as theories which might be discarded *ad libitum*, little heeding that there is no science which subsists upon induction alone, this being only the necessary understructure of the building. Says Mill (*Logic*, p. 286): "A revolution is peaceably and progressively effecting itself in philosophy, the reverse of that to which Bacon has attached his name. That great man changed the method of the sciences from deductive to experimental, but it is now rapidly reverting from experimental to deductive. Deduction is the great scientific work of the present and future ages."

Half a century before this was written Hahnemann gave his science complete to the world, just as Minerva came all armed and grown up from Jupiter's brain. By careful induction from well-observed facts, he arrived at the principle of Homœopathy, from which inversely the laws for the facts of induction were deduced, and his deductions find their verifications in the daily practice of homœopathicians with high potencies.

Let us now proceed to his induction as the verification of his deduction given above.

Hahnemann, after an experience of forty-three years, and a Hahnemannian experience at that, declares (sec. 279) that "the dose of the homœopathically selected remedy can never be prepared so small that it should not be still stronger than the natural disease, and potent enough to at least in part overcome, extinguish, and cure it as long as it is able to, immediately after the administration, cause some, though slight, preponderance of its symptoms over the disease similar to it."

He himself has proved the thirtieth centesimal potency upon the healthy, and the result of his provings is laid down in his four volumes of *Chronic Diseases*, which is only a continuation of his *Materia Medica Pura* in six volumes. Hahnemann continues (sec. 128): "Thus we now explore in the best manner even the substances deemed to be weak with regard to their medicinal power, if we let the prover take daily four to six pellets of the thirtieth (centesimal) potentiated attenuation of such a substance moistened with a little water, and continue this for several days."

So this may be put down as an element of induction, that he has actually proved the thirtieth centesimal potency upon the healthy in numerous instances, and found them producing symptoms of disease available for the cure of similar ailments.

Since then the potentiation of medicinal substances has been carried higher and higher, so that what has been predicated by Hahnemann in regard to the thirtieth centesimal holds equally true with regard to the five millionth centesimal, of which an undoubted proving has been given by Dr. Buchmann, see HOMŒOPATHIC PHYSICIAN, Vol. III, p. 260. And since writing this, a new proving of Lachesis 6M by the same observer has come to hand, so that the reliable scale of potentiation extends now to the six millionth centesimal potency.

The next element of induction is in the application of the proper homœopathic remedy upon the sick (sec. 148). "A remedy thus selected, which has the power and tendency to excite the symptoms most possibly similar to the disease to be cured, therefore a similar artificial disease, in its dynamical action upon the morbidly distuned vital force of man seizes in proper dose just those parts and points in the organism hitherto suffering from the natural disease, and excites in them their own artificial disease, which then, chiefly on account of the great simility and surpassing strength, steps in the place of the so far natural distunement of disease, so that the instinctive automatic vital force from this moment suffers no more from the natural but from the stronger similar medicine-disease which then again, on account of the small dose of the remedy like every moderate medicine-disease, conquered by the heightened energy of the vital force, soon vanishes by itself, and leaves the body free from all disease, *i. e.*, sound and permanently healthy." (Compare sec. 28).

(Sec. 275.) "The adaptedness of a medicine to a given state of disease depends not alone upon its striking homœopathic selection, but just as well upon the requisite correct magnitude, or rather minuteness, of its dose."

(Sec. 281.) "Every patient, particularly in point of his disease, is incredibly liable to be affected by the medicinally powerful potencies proportionate by the simility of action; and there is no man, be he ever so robust, even when afflicted with a chronic or so-called local disease, who would not soon feel the most desired improvement in the suffering part after having taken the helpful homœopathically adapted medicine in the smallest dose to be thought of; who, in one word, should no more be affected in his system than the nursling only one day old is changed by it. How insignificant and ridiculous, therefore, is this theoretical unbelief against these never-failing, unerring proofs of experience."

No more need be said to point out the facts upon which these sections from the *Organon* are based. They are before the world in the innumerable cures which the homœopathicians have performed by those Hahnemannian potencies; and it is the most prominent and gifted of our profession who have given and give yet their unqualified testimony in favor of the highly potentiated dose of the drug.

Dr. P. P. Wells, in his address, "Philosophy of Medicine" (HOMŒOPATHIC PHYSICIAN, Vol. I, p. 281), says: "The claim to our acceptance of the dynamized dose in our practical duties rests, then, wholly on the testimony of those who have tried it, and who know the truth of what they affirm. The history of the dynamized dose, as contained in the testimony of these witnesses, declares its superior curative power as compared with doses of crude drugs. It should be borne in mind, when considering the statements of those witnesses, that most of them are the more competent to give us testimony worthy of our regard for the reason that they have practically tried both kinds, and have found greater curing power in that which is dynamized."

This declaration of the superior power of potentiated medicine by this trustworthy authority could not have been made without having the sure foundation of "pure experiments, careful observation, and correct experience" (*Organon*, sec. 278), which is to be found in our literature and confirmed daily and hourly in the practice of every homœopathician. The Hahnemannian postulatum of the necessity of potentiation of the remedy has been verified for potencies as high as five million centesimally in two cases related by Dr. P. P. Wells in his article, "Routine Practice." (See HOMŒOPATHIC PHYSICIAN, Vol. III, pp. 289, 291.)

The position taken in the foregoing quotation, moreover, is strengthened by the reflection that what here has been maintained on the ground of experiment and observation alone is also confirmed by the general principle enunciated by Hahnemann as above stated. In fact, it was this experience which caused him to pronounce it *in extenso* in the first part of the *Organon*.

Here, then, is the second element of induction—the successful application of the proper potentiated dose of the homœopathic remedy in disease.

We now proceed to the third element, which is the potentiation of substances used as medicine. Hahnemann went higher and higher till he died. Korsakoff, before Hahnemann's decease, had taken up the subject and opened a new vista, which in his time was approved of by Hahnemann himself. Korsakoff went as high as 1,500. After Korsakoff Jenichen sacrificed himself for this great cause, reaching as high as the 40m. After him the writer arrived at 6M centes., and found the end of medicinal action not yet, as the above-mentioned provings and clinical cases prove. Whether there is an end at all the experiment will show by and by. The data given are sufficient, however, to establish the fact that the high potencies DO act, and raise the question: How can there be any medicinal power in a six millionth centesimal potency? The answer requires an extensive disquisition which would surpass the limits of this paper, and there is doubt whether it would satisfy the questioner—for a child may ask a question which the wisest man cannot answer. Let us, therefore, avoid the, for our purpose, idle question, and rather ask, if there is medicinal power in a six millionth centesimal, what follows from it for practical use? a proceeding which amounts to the Baconian method of induction without which we always will get into a Slough of Despond. There is this fact, that a palpable substance which we perfectly know has been brought in contact with an immense mass of inert vehicle six millions of times in the proportion of one to hundred. There is the other fact, that such a distribution of its medicinal force to the five millionth centesimal potency acts upon the human organism both in health and disease. There is another fact, that the characteristic medicinal action of the substance from which the potentiation had been started is clearly discernible in the proving and healing cases. Thus the identity of the substance is proved through the whole series of six million potencies. There is another fact, that the chronoscopic and electro-magnetic neural analysis proves the potentiality of such high potencies by the visible demonstration of the nerve system.

And these facts form the third element of our induction, and prove conclusively that it cannot possibly be the matter of the substance used for potentiation which is exerting its force upon the living organism, but it must be something else, for which we have no other means of finding a reasonable explanation than the analogy of the spiritual and psychological facts which likewise cannot be referred to the operation of matter upon matter.

There need be no faltering on the part of the homœopathician on account of the dicta of the natural sciences. They have presumed too much in one way, and yielded too much in the opposite way. They have declared that spiritual matters transcend their horizon, as naturally they should, and have raised their ignorance to the dignity of a principle. No light can be expected from that quarter for our practical questions: What follows from the homœopathic fact of potentiation? In what relation does it stand to the general law of homœopathics?

It follows that there must be something in the body of man which is able to be affected by such immaterial potencies as are produced by potentiation of palpable substances. This something can only be the nerve system, because the application of the potencies produces different symptoms according to the direction of its specific relation to the organism, just as the slight galvanic current in the electro-magnetic analysis shows a varying resistance on the part of the nerve system. It follows that since the impression of the immaterial potency upon the nerve terminations cannot be a material one because no matter is concerned in it, there must be a system standing beyond the nerve system in intimate connection with it, and in continuous control of it through life, by which the functions are regulated, acting similarly as the medicinal potency, not by material processes, but in a spirit-like manner. This something is what Hahnemann terms the vital force. Hence it might also properly be termed the nerve-spirit, and it would be a legitimate physiological designation, as the combination of the physiological functions or the resultant of the vital forces of the parts of the organism. This is what Hahnemann admirably described in sec. 9: "In health the spirit-like force (autocracy) which as dynamis animates the material body rules absolutely and holds all its parts in admirably harmonic course of life in sensations and activities, in such a manner that our indwelling rational spirit can use freely this living sound instrument for the higher ends of our being."

Thus by the strict inductive method of Hahnemann we, of necessity, are driven to acknowledge the existence of an immaterial power called the vital force (nerve-spirit), from which Hahnemann deduced the laws of the homœopathic science, viz.:

(1.) The law of *Similia similibus curantur.*

(2.) The law of the properly potentiated dose; and they are so intertwined by their logical interdependence upon one another that they cannot be separated without jeopardizing the truth and doing injury to common sense.

If there would be no *Similia* there would have been no potentiation. As soon as Hahnemann discovered that it was the vital force which substitutes the pathopoësis to the pathogenesis, he needed a remedy which would disappear in the substitutive process, and this could only be one which was similar in its utterance, though different in kind (sec. 26) viz.: an immaterial remedy which he very appropriately styled a potency. It does not weaken the force of the argument that his mode of arriving at the discovery of potentiation was experiential and experimental; on the contrary, it can only strengthen the Hahnemannian position, because it led him to the wise demonstration of the healing process by the inductive method, and in this scientific way was found that true reason of the homœopathic law according to the mathematical and philosophical postulatum for comparison, that things can only be compared if of the same kind. It does not matter that we here have to deal with psychological realities which are immaterial; they are as real in their way as any material things subject to gravitation. These realities are here on one side the spirit-like action of the vital force, and on the other side the spirit-like action of the potency. They, certainly, are of the same kind in regard to the spirituality of their action, though different, inasmuch as the vital force belongs to the organism over which it exerts its control, and the potency to the substance from which it has been abstracted. The *Similia* could not be known except by the action upon the vital force. We merely observe symptoms from disease, and these symptoms are the result of the negative change of the vital force by the positive action of potencies known and unknown.

But if Hahnemann had not discovered Homœopathy we would not know that these symptoms are *Similia* of similar changes of the vital force, brought about by potencies developed homœopathically from well-known substances. Now by the homœopathic application we know that these potencies are the opposite positive *Similia*, which when brought in opposite action by the administration neutralize the opposed *Similia* of the body by substitution and the over-action of the *Least Plus* of the potencies, and restore the negative change of the vital force to its neutral and normal condition of health.

Consequently, the position taken in this *Thesis* is in full accord with the general law pronounced by Hahnemann in sec. 26, and it follows necessarily that the law of *Similia* is a consequence of potentiation, for without potentiation there would be no *Similia*, and potentiation is a consequence of *Similia*, for without *Similia* there would be no potentiation.

*Ceterum censeo, macrodosiam esse delendam.*

BROOKLYN, April 19th, 1884.

---

THE GOLDEN RULE.—Do as you would be done by! pay your subscriptions promptly as you would have your patients pay you.

